# Sleep and brain, physical and societal health

**DOI:** 10.1192/j.eurpsy.2025.79

**Published:** 2025-08-26

**Authors:** E. Pajediene

**Affiliations:** Neurology Department, Lithuanian University of Health Sciences, Kaunas, Lithuania

## Abstract

**Abstract:**

Sleep is essential for physical, brain (including mental) and societal health. Sleep-wake disorders are confirmed as independent risk factors and/or modulators of several neurological (such as stroke, dementia, and parkinsonism), psychiatric (depression) and other (arterial hypertension, diabetes, oncological) disorders. According to the Cost Of Illness in Neurology in Europe (COIN-EU) Study, 1.7 trillion euros cost is estimated for neurological disorders, of which 25.45% is dedicated for sleep disorders. In this talk both the socioeconomic burden of sleep disorders, and possible sleep-based interventions for improving brain and physical health, will be discussed.

**Disclosure of Interest:**

None Declared

